# A pragmatic randomized trial evaluating pre-operative aqueous antiseptic skin solutions in open fractures (Aqueous-PREP): statistical analysis plan

**DOI:** 10.1186/s13063-022-06541-0

**Published:** 2022-09-12

**Authors:** Nathan N. O’Hara, Diane Heels-Ansdell, Sofia Bzovsky, Shannon Dodds, Lehana Thabane, Mohit Bhandari, Gordon Guyatt, P. J. Devereaux, Gerard P. Slobogean, Sheila Sprague, on behalf of the PREP-IT Investigators, Anthony D. Harris, C. Daniel Mullins, Jeffrey Wells, Amber Wood, Gregory J. Della Rocca, Joan Hebden, Kyle J. Jeray, Lucas S. Marchand, Lyndsay M. O’Hara, Robert Zura, Christopher Lee, Joseph Patterson, Michael J. Gardner, Jenna Blasman, Jonah Davies, Stephen Liang, Monica Taljaard, Lehana Thabane, Debra Marvel, Jana Palmer, Jeff Friedrich, Frances Grissom, I. Leah Gitajn, Saam Morshed, Robert V. O’Toole, Bradley A. Petrisor, Franca Mossuto, Manjari G. Joshi, Jean-Claude D’Alleyrand, Justin Fowler, Jessica Rivera, Max Talbot, Sheila Sprague, David Pogorzelski, Silvia Li, Alejandra Rojas, Gina Del Fabbro, Olivia Paige Szasz, Paula McKay, Alexandra Minea, Kevin Murphy, Andrea Howe, Haley K. Demyanovich, Michelle Medeiros, Genevieve Polk, Eric Kettering, Nirmen Mahal, Andrew Eglseder, Aaron Johnson, Christopher Langhammer, Christopher Lebrun, Jason Nascone, Raymond Pensy, Andrew Pollak, Marcus Sciadini, Yasmin Degani, Heather Phipps, Eric Hempen, Herman Johal, Bill Ristevski, Dale Williams, Matthew Denkers, Krishan Rajaratnam, Jamal Al-Asiri, Jodi Gallant, Kaitlyn Pusztai, Sarah MacRae, Sara Renaud, John D. Adams, Michael L. Beckish, Christopher C. Bray, Timothy R. Brown, Andrew W. Cross, Timothy Dew, Gregory K. Faucher, Richard W. Gurich, David E. Lazarus, S. John Millon, M. Jason Palmer, Scott E. Porter, Thomas M. Schaller, Michael S. Sridhar, John L. Sanders, L. Edwin Rudisill, Kyle M. Altman, Julia C. Quirion, Markus F. Loeffler, Erin R. Pichiotino, Austin A. Cole, Ethan J. Maltz, Wesley Parker, T. Bennett Ramsey, Alex Burnikel, Michael Colello, Russell Stewart, Jeremy Wise, M. Christian Moody, Matthew Anderson, Joshua Eskew, Benjamin Judkins, James M. Miller, Stephanie L. Tanner, Rebecca G. Snider, Emily Bray, Harper Abbott, Roman M. Natoli, Todd O. McKinley, Walter W. Virkus, Anthony T. Sorkin, Jan P. Szatkowski, Brian H. Mullis, Yohan Jang, Luke A. Lopas, Lauren C. Hill, Courteney L. Fentz, Maricela M. Diaz, Krista Brown, Katelyn M. Garst, Emma W. Denari, Patrick Osborn, Sarah Pierrie, Maria Herrera, Theodore Miclau, Meir Marmor, Amir Matityahu, R. Trigg McClellan, David Shearer, Paul Toogood, Anthony Ding, Jothi Murali, Ashraf El Naga, Jennifer Tangtiphaiboontana, Tigist Belaye, Eleni Berhaneselase, Dmitry Pokhvashchev, William T. Obremskey, Amir Alex Jahangir, Manish Sethi, Robert Boyce, Daniel J. Stinner, Phillip Mitchell, Karen Trochez, Elsa Rodriguez, Charles Pritchett, Natalie Hogan, A. Fidel Moreno, Jennifer E. Hagen, Matthew Patrick, Richard Vlasak, Thomas Krupko, Michael Talerico, Marybeth Horodyski, Chris Koenig, Marissa Pazik, Elizabeth Lossada-Soto, Joshua L. Gary, Stephen J. Warner, John W. Munz, Andrew M. Choo, Timothy S. Achor, Milton L. Chip Routt, Michael Kutzler, Sterling Boutte, Ryan J. Warth, Michael Prayson, Indresh Venkatarayappa, Brandon Horne, Jennifer Jerele, Linda Clark, Christina Boulton, Jason Lowe, John T. Ruth, Brad Askam, Andrea Seach, Alejandro Cruz, Breanna Featherston, Robin Carlson, Iliana Romero, Isaac Zarif, Niloofar Dehghan, Michael McKee, Debra L. Sietsema, Alyse Williams, Tayler Dykes, Ernesto Guerra-Farfan, Jordi Tomas-Hernandez, Jordi Teixidor-Serra, Vicente Molero-Garcia, Jordi Selga-Marsa, Juan Antonio Porcel-Vazquez, Jose Vicente Andres-Peiro, Joan Minguell-Monyart, Jorge Nuñez-Camarena, Maria del Mar Villar-Casares, Jaume Mestre-Torres, Pilar Lalueza-Broto, Felipe Moreira-Borim, Yaiza Garcia-Sanchez, Francesc Marcano-Fernández, Laia Martínez-Carreres, David Martí-Garín, Jorge Serrano-Sanz, Joel Sánchez-Fernández, Matsuyama Sanz-Molero, Alejandro Carballo, Xavier Pelfort, Francesc Acerboni-Flores, Anna Alavedra-Massana, Neus Anglada-Torres, Alexandre Berenguer, Jaume Cámara-Cabrera, Ariadna Caparros-García, Ferran Fillat-Gomà, Ruben Fuentes-López, Ramona Garcia-Rodriguez, Nuria Gimeno-Calavia, Marta Martínez-Álvarez, Patricia Martínez-Grau, Raúl Pellejero-García, Ona Ràfols-Perramon, Juan Manuel Peñalver, Mònica Salomó Domènech, Albert Soler-Cano, Aldo Velasco-Barrera, Christian Yela-Verdú, Mercedes Bueno-Ruiz, Estrella Sánchez-Palomino, Vito Andriola, Matilde Molina-Corbacho, Yeray Maldonado-Sotoca, Alfons Gasset-Teixidor, Jorge Blasco-Moreu, Núria Fernández-Poch, Josep Rodoreda-Puigdemasa, Arnau Verdaguer-Figuerola, Heber Enrique Cueva-Sevieri, Santiago Garcia-Gimenez, Darius G. Viskontas, Kelly L. Apostle, Dory S. Boyer, Farhad O. Moola, Bertrand H. Perey, Trevor B. Stone, H. Michael Lemke, Ella Spicer, Kyrsten Payne, Robert A. Hymes, Cary C. Schwartzbach, Jeff E. Schulman, A. Stephen Malekzadeh, Michael A. Holzman, Greg E. Gaski, Jonathan Wills, James S. Ahn, Sharmistha Das, Antoinisha D. English, Jaslynn A. N. Cuff, Holly Pilson, Eben A. Carroll, Jason J. Halvorson, Sharon Babcock, J. Brett Goodman, Martha B. Holden, Wendy Williams, Taylor Hill, Ariel Brotherton, Nicholas M. Romeo, Heather A. Vallier, Joanne Fraifogl, Anna Vergon, Thomas F. Higgins, Justin M. Haller, David L. Rothberg, Ashley Neese, Zachary M. Olsen, Abby V. McGowan, Sophia Hill, Morgan K. Dauk, Patrick F. Bergin, George V. Russell, Matthew L. Graves, John Morellato, Sheketha L. McGee, Eldrin L. Bhanat, Ugur Yener, Rajinder Khanna, Priyanka Nehete, David Potter, Robert VanDemark, Kristi Atkins, Marcus Coe, Kevin Dwyer, Devin S. Mullin, Theresa A. Chockbengboun, Kevin Phelps, Michael Bosse, Madhav Karunakar, Laurence Kempton, Stephen Sims, Joseph Hsu, Rachel Seymour, Christine Churchill, Ada Mayfield, Juliette Sweeney, Todd Jaeblon, Robert Beer, Brent Bauer, Sean Meredith, Caroline Benzel, Christopher M. Domes, Rachel M. Reilly, Ariana Paniagua, Ja Nell Dupree, Michael J. Weaver, Arvind G. von Keudell, Abigail E. Sagona, Samir Mehta, Derek Donegan, Annamarie Horan, Mary Dooley, Marilyn Heng, Mitchel B. Harris, David W. Lhowe, John G. Esposito, Ahmad Alnasser, Steven F. Shannon, Alesha N. Scott, Bobbi Clinch, Becky Weber, Michael J. Beltran, Michael T. Archdeacon, Henry Claude Sagi, John D. Wyrick, Theodore Toan Le, Richard T. Laughlin, Cameron G. Thomson, Kimberly Hasselfeld, Carol A. Lin, Mark S. Vrahas, Charles N. Moon, Milton T. Little, Geoffrey S. Marecek, Denice M. Dubuclet, John A. Scolaro, James R. Learned, Philip K. Lim, Susan Demas, Arya Amirhekmat, Yan Marco Dela Cruz

**Affiliations:** 1 R Adams Cowley Shock Trauma Center, Department of Orthopaedics, University of Maryland School of Medicine, 22 South Greene Street, Baltimore, MD USA; 2 Department of Health Research Methods, Evidence, and Impact, McMaster University, Hamilton, Ontario Canada; 3 Division of Orthopaedic Surgery, Department of Surgery, McMaster University, Hamilton, Ontario Canada; 4 Department of Medicine, McMaster University, Hamilton, Ontario Canada; 5 Population Health Research Institute, McMaster University, Hamilton, Ontario Canada

**Keywords:** Open fracture, Surgical site infection, Aqueous antiseptic solutions

## Abstract

**Background:**

Approximately 1 in 10 patients with a surgically treated open fracture will develop a surgical site infection. The Aqueous-PREP trial will investigate the effect of 10% povidone-iodine versus 4% chlorhexidine in aqueous antiseptic solutions in reducing infections after open fracture surgery. The study protocol was published in April 2020.

**Methods and design:**

The Aqueous-PREP trial is a pragmatic, multicenter, open-label, randomized multiple period cluster crossover trial. Each participating cluster is randomly assigned in a 1:1 ratio to provide 1 of the 2 study interventions on all eligible patients during a study period. The intervention periods are 2 months in length. After completing a 2-month period, the participating cluster crosses over to the alternative intervention. We plan to enroll a minimum of 1540 patients at 14 sites.

**Results:**

The primary outcome is surgical site infection guided by the Centers for Disease Control and Prevention’s National Healthcare Safety Network reporting criteria (2017). All participants’ surgical site infection surveillance period will end 30 days after definitive fracture management surgery for superficial infections and 90 days after definitive fracture management surgery for deep incisional or organ/space infections [1]. The secondary outcome is an unplanned fracture-related reoperation within 12 months of the fracture.

**Conclusion:**

This manuscript serves as the formal statistical analysis plan (version 1.0) for the Aqueous-PREP trial. The statistical analysis plan was completed on February 28, 2022.

## Administrative information

**Table:** 

Title	A Pragmatic Randomized trial Evaluating Pre-operative aqueous antiseptic skin solutions in open fractures (Aqueous-PREP)
**Trial Registration**	clinicaltrials.gov, NCT03385304. Registered December 28 2017, https://www.clinicaltrials.gov/ct2/show/NCT03385304?term=slobogean&draw=2&rank=3
**SAP Version**	1.0
**Protocol Version**	2.1
**SAP Revisions**	None

## Introduction

### Background and rationale

The prevention of infection is a critical goal of perioperative care for patients with surgically treated open fractures. Surgical site infections are often devastating complications for open fracture patients because of the unplanned reoperations, fracture healing difficulties, and adverse events from prolonged antibiotic treatments. Given the severity of open fractures, maximizing the effectiveness of current prophylactic procedures is essential.

Standard practice in the management of open fractures includes cleaning the injured limb with an antiseptic skin solution in the operating room prior to making a surgical incision. The available solutions kill bacteria and decrease the quantity of native skin flora, thereby reducing surgical site infection [2–5]. While there is extensive guidance on specific procedures for prophylactic antibiotic use and standards for sterile technique, the evidence regarding the choice of antiseptic skin preparation solution is very limited for open fracture surgery.

The Aqueous-PREP trial will provide the necessary evidence to guide the choice of antiseptic skin solution to prevent surgical site infections in patients with open fractures. The trial is poised to significantly impact the care and outcomes of open extremity fracture patients.

### Objectives

The overall objective of the Aqueous-PREP trial is to compare the effect of 10% povidone-iodine versus 4% chlorhexidine in aqueous antiseptic solutions for the surgical management of open fractures.

#### Primary objective and hypothesis

To determine the effect of 10% povidone-iodine versus 4% chlorhexidine in aqueous antiseptic solutions in preventing surgical site infections. We hypothesize that 10% povidone-iodine aqueous antiseptic will be more effective in preventing surgical site infections than 4% chlorhexidine aqueous antiseptic [5, 6].

#### Secondary objective and hypothesis

To determine the effect of 10% povidone-iodine versus 4% chlorhexidine in aqueous antiseptic solutions in preventing unplanned fracture-related reoperations. We hypothesize that 10% povidone-iodine aqueous antiseptic will be more effective in preventing unplanned reoperations than 4% chlorhexidine aqueous antiseptic [5, 6].

#### Subgroup objectives and hypotheses

We will perform 3 subgroup analyses to determine if the effects of preoperative antiseptic skin solutions on surgical site infection vary within clinically relevant subgroups. The primary subgroup will be defined by the severity of the open fracture. Secondary subgroups will include the location of the fracture and the severity of wound contamination. We hypothesize that the magnitude of the effect of 10% povidone-iodine aqueous antiseptic compared with 4% chlorhexidine aqueous antiseptic in preventing surgical site infections will be greater in Gustilo-Anderson type III open fractures versus Gustilo-Anderson type I or II open fractures [7], lower extremity fractures versus upper extremity fractures, and wounds with embedded contamination versus wounds with no, minimal, or surface contamination according to the Orthopedic Trauma Association Open Fracture Classification (OTA-OFC) [8–10].

### Reporting

The structure of this statistical analysis plan follows the Guidelines for the Content of Statistical Analysis Plans in Clinical Trials [11]. The reporting of the trial results will follow the 2010 CONSORT statement and the extension statements for Cluster Trials and Randomized Crossover Trials, as applicable [12]. Additional statistical analyses plans will be developed for secondary analyses of the trial data.

## Study methods

### Trial design

The study is a pragmatic, multicenter, open-label, randomized multiple period cluster crossover trial. We defined clusters as orthopedic practices within participating hospitals, with each participating hospital having only one participating orthopedic practice [13]. The intervention periods are approximately 2 months in length. After completing a 2-month period, the participating cluster crosses over to the alternative intervention where they use the other study solution for the next 2-month period. There are no washout periods between treatment periods.

### Randomization

The order of treatment allocation for each orthopedic practice (cluster) will be randomly assigned using a computer-generated randomization table. Each cluster will start with the initially allocated study solution and crossover to the other solution for their second recruitment period. This process of alternating treatments will repeat approximately every 2 months as dictated by the initial randomization until enrollment targets are met. The randomization will be in a 1:1 ratio, unrestricted, and executed only prior to the first sequence.

### Sample size

A sample size of 1540 patients will have 80% power to detect a 38% reduction in the odds of infection with a two-sided alpha of 0.05. This estimate allows for a 10% loss to follow-up and assumes a baseline infection risk of 12.5%, 10 recruiting clusters, no between-period variance, and a 0.095 between-cluster variance [6]. After the initial power calculations, we determined that additional clusters were required to meet the study timelines. As such, we increased the number of clusters from 10 to 14. The increase in clusters results in a marginal increase in statistical power (approximately 2%).

### Framework

All study outcomes will be tested for superiority.

### Interim analysis and stopping guidance

Aqueous-PREP does not have a planned interim analysis. However, the trial’s Data and Safety Monitoring Committee reviews the reporting of serious adverse events biannually and can recommend early stopping if safety concerns are identified.

### Timing of outcome assessments

Research personnel will contact study participants at 6 weeks, 3 months, 6 months, 9 months, and 12 months after their fracture. Our primary outcome will be surgical site infection (SSI) and it will be assessed at 30 days (superficial infections) and at 90 days (deep and organ space infections) after definitive fracture management surgery. The secondary outcome will be occurrence of an unplanned fracture-related reoperation within 12 months of the fracture. Additional time points will be used for our planned sensitivity analyses.

## Statistical principles

### Confidence intervals and *P*-values

All statistical tests will be two-sided and performed using a 5% significance level. We will report all confidence intervals as 95% and two-sided. All results will be expressed as odds ratios produced by analysis described in section 5.2. Interaction *p*-values will be provided for the subgroup analyses. We will not adjust for multiple testing, and all sensitivity analyses and secondary results will be interpreted as exploratory.

### Adherence and protocol deviations

Adherence will be assessed at the definitive fracture surgery for each participant and will be binary in its definition. We will report adherence as the number and percentage of participants who received the allocated intervention at their definitive fracture management surgery. We will also tabulate the reasons for non-adherence. The adherence percentages and reasons for non-adherence will be reported by treatment arm.

Our rationale for defining adherence based solely on the antiseptic solution used during the definitive fracture management surgery is two-fold. (1) The definitive fracture management surgery involves the final implantation of the surgical fixation hardware, when it is most susceptible to bacterial contamination and biofilm development. (2) Any open fracture surgeries prior to the definitive fracture management surgery are staged procedures to remove gross contamination, temporarily stabilize fractures in multi-trauma patients, and minimize evolving soft tissue injuries. Temporally, these procedures occur prior to the surgery of interest for the trial’s objectives, and if bacterial contamination had occurred in one of the proceeding procedures, the repeat surgical debridement and perioperative antibiotics would reduce the likelihood of persistent occult infection occurring prior to the definitive fracture surgery.

### Analysis populations

#### Intention-to-treat

Our primary analysis will use the intention-to-treat approach and will include all enrolled patients in the treatment groups to which their cluster was allocated at the time of their first fracture management surgery.

#### As-treated

One of our sensitivity analyses will be performed on an as-treated population (see the “Sensitivity analyses” section). The as-treated population will include participants from the intention-to-treat population but classified based on the intervention received at their definitive fracture management surgery. Participants who do not receive one of the two study interventions will be excluded from this analysis. This approach for defining the as-treated treatment groups is a simpler adaptation of what was initially proposed in the protocol. This final approach was selected to be consistent with the classification of adherence outlined above.

## Trial population

### Cluster screening and eligibility

Prior to commencing the trial, the investigators solicited orthopedic surgery practices treating open fracture patients in hospitals in the USA, Canada, and Spain to participate in the trial. All potential clusters completed a feasibility questionnaire prior to initiating start-up activities. To be included in the trial, each cluster had to demonstrate: (1) adequate research personnel infrastructure to manage the study, (2) adequate fracture patient volume to complete enrolment within the study timeline, (3) a commitment from all surgeons to adhere to the assigned interventions, and (4) the ability to procure both study interventions. All hospitals started with a run-in phase of at least 1 month to demonstrate that they could adhere to the trial protocol prior to commencing the study.

We will report the number of clusters (orthopedic practices) screened, included, and excluded in a flow diagram. The number of clusters excluded by reason has been reported previously [13]. Cluster randomization allocation will be included in the flow diagram, and adherence with treatment allocation during the run-in period by cluster will be summarized using percentages.

### Patient screening and eligibility

All patients 18 years of age or older who present to a recruiting hospital for treatment of an open fracture(s) of the appendicular skeleton will be screened by a research staff member for participation within 3 weeks of their fracture. Eligible patients must receive surgical debridement of their open fracture wound(s) within 72 h of their injury, and the open fracture(s) must be managed definitively with a surgical implant (e.g., internal fixation, external fixation, joint prosthesis). Written informed consent is required for study enrollment to permit the clinical follow-up of study participants. However, our institutional review board did not require informed consent to occur prior to the study treatment, given the urgent nature of the surgery and the predetermination of the two commonly used interventions with cluster-crossover design. The patients, treating clinicians, and research team members at the participating sites are unmasked to the treatment allocation.

**Fig. 1:**
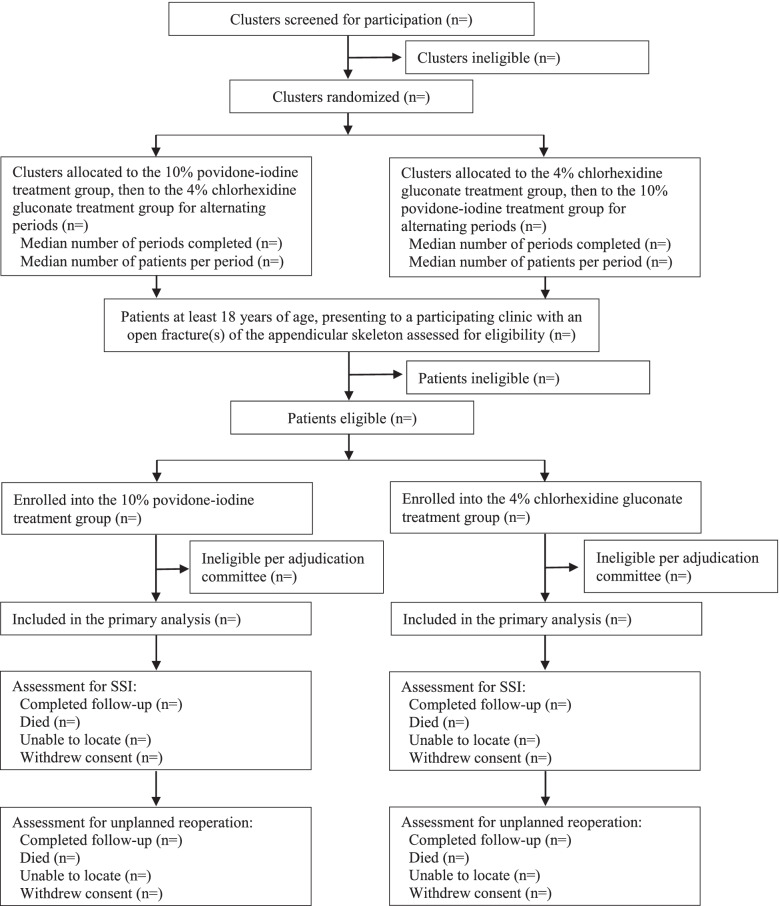
CONSORT Flowchart for Participants

The number of patients screened, included, and excluded will be presented in a flow diagram (Fig. 1). The figure will consist of the number of patients who were eligible, ineligible, and enrolled. In addition, the number of patients excluded by reason will be summarized**.** We will also list the number of participants who were enrolled and subsequently deemed ineligible by the Central Adjudication Committee by treatment group and overall. Participants deemed ineligible by a Central Adjudication Committee blinded to the treatment will not be included in any analysis, as per the guidance of Fergusson et al. [14].

### Participant withdrawal

The level of withdrawal will be tabulated and classified as “withdrawal of consent” or “lost to follow-up.” Participant deaths will also be tabulated.

### Participant follow-up

We will report the number of participants who complete follow-up at 3 months after definitive fracture management surgery and 12 months after their fracture, stratified by treatment allocation.

### Cluster characteristics

Specific details on characteristics of participating clusters, orthopedic characteristics, and surgical infection prevention information in the Aqueous-PREP trial have been previously published [13].

### Participant demographics, fracture characteristics, and descriptions of surgical and perioperative care

We will describe the study population with respect to age, sex, body mass index, diabetes status, smoking status, Injury Severity Score, the severity of the open fracture according to the Gustilo-Anderson classification [7], the location of the fracture, level of wound contamination using the OTA-OFC classification [8], the American Society of Anesthesiologists Physical Status Classification System, the use of temporary fracture stabilization, the use of intraoperative topical antibiotics, and the duration of perioperative antibiotic administration. We also will report the use of antiseptic preoperative bathing and the method of wound closure (Table 1). Categorical data will be summarized by counts with percentages. Age will be summarized as a mean with standard deviation. We will report the Injury Severity Score as a median with an interquartile range. The duration of systemic perioperative antibiotics will be summarized in days and reported as a median with interquartile range. Body mass index (BMI) will be reported in kg/m^2^ and subcategorized as underweight (BMI < 18.5), normal weight (18.5–24.9), overweight (25–29.9), and obese (BMI > 30). Additional patient characteristics may be reported as supplemental information. All reporting will be stratified by treatment groups. We will not statistically test for differences in baseline characteristics between treatment groups; however, the clinical importance of any imbalance will be noted.

**Table 1:** Baseline characteristics and fracture management

	Povidone-Iodine	Chlorhexidine
*Participant characteristics*	**(*n =*** **XXX)**	**(*n =*** **XXX)**
Age, years, mean (SD)		
Sex, *n* (%)		
Male		
Body mass index, kg/m^2^, *n* (%)		
Underweight (BMI < 18.5)		
Normal weight (18.5–24.9)		
Overweight (25–29.9)		
Obese (BMI > 30)		
Diabetes, *n* (%)		
Current smoker, *n* (%)		
Injury severity score, median (IQR)		
American Society of Anesthesiologists Physical Score, *n* (%)		
Class I or II		
Class III or higher		
*Fracture characteristics*^*†*^	**(*n =*** **XXX)**	**(*n =*** **XXX)**
Severity of open fracture, *n* (%)		
Gustilo-Anderson type I/II		
Gustilo-Anderson type IIIA		
Gustilo-Anderson type IIIB/IIC		
Location of fracture, *n* (%)		
Lower extremity or pelvis		
Upper extremity		
Wound contamination, *n* (%)		
None or minimal contamination		
Surface contamination		
Contaminant embedded in bone or deep soft tissue		
*Fracture management*^*†*^	**(*n =*** **XXX)**	**(*n*** **= XXX)**
Temporary fracture stabilization, *n* (%)		
Number of planned surgeries, *n* (%)		
1		
2		
3 or more		
Preoperative antiseptic bathing with chlorhexidine gluconate^*^, *n* (%)		
Method of definitive fracture management, *n* (%)		
Plate and screw fixation		
Intramedullary nail		
External fixation		
Other		
Intraoperative topical antibiotics^*^, *n* (%)		
Skin closure method^**^, *n* (%)		
Primary wound closure		
Skin graft		
Local flap		
Free flap		
No closure attempted/secondary wound healing		

^*†*^Fracture data includes all eligible participant injuries

^*^Intervention reported for the definitive fracture management surgery only

^**^Only the most complex wound closure method is reported if multiple methods were used

## Analysis

### Outcome definitions

#### Primary outcome

Our primary outcome is SSI guided by the Centers for Disease Control and Prevention’s (CDC) National Healthcare Safety Network reporting criteria (2017) [1]. The SSI surveillance period for all participants, including participants with multiple planned fracture surgeries, will end 30 days after definitive fracture management surgery for superficial SSI and 90 days after definitive fracture management surgery for deep incisional or organ/space SSI. We will also separately report but not statistically test the occurrence of each type of SSI (superficial incisional infections by 30 days, deep incisional infections by 90 days, and organ/space infections by 90 days) by treatment arm. If multiple tissue levels are involved in the infection, the type of SSI will be defined by the deepest tissue layer involved during the surveillance period. Therefore, only one type of SSI per participant will be reported.

**CDC National Healthcare Safety Network Surgical Site Infection Reporting Criteria (2017).**

**Table:** 

Outcome	Description
*Superficial Incisional SSI*	Date of event for infection may occur from the date of fracture to 30 days after the definitive fracture management surgery **AND** involves only skin and subcutaneous tissue of the incision **AND** patient has at least one of the following: a. purulent drainage from the superficial incision. b. organisms identified from an aseptically obtained specimen from the superficial incision or subcutaneous tissue by a culture or non-culture based microbiologic testing method which is performed for purposes of clinical diagnosis or treatment (e.g., not Active Surveillance Culture/Testing [ASC/AST]). c. superficial incision that is deliberately opened by a surgeon, attending physician or other designee and culture or non-culture-based testing is not performed. **AND **patient has at least one of the following signs or symptoms: pain or tenderness; localized swelling; erythema; or heat. d. diagnosis of a superficial incisional SSI by the surgeon or attending physician or other designee. The following do not qualify as criteria for meeting the definition of superficial SSI: •Diagnosis/treatment of cellulitis (redness/warmth/swelling), by itself, does not meet criterion “d” for superficial incisional SSI. Conversely, an incision that is draining or that has organisms identified by culture or non-culture-based testing is not considered a cellulitis. •A stitch abscess alone (minimal inflammation and discharge confined to the points of suture penetration). •A localized stab wound or pin site infection- Such an infection might be considered either a skin (SKIN) or soft tissue (ST) infection, depending on its depth, but not an SSI Note: A laparoscopic trocar site for an operative procedure is not considered a stab wound. •An infected burn wound is classified as BURN and is not an SSI.
*Deep Incisional SSI*	The date of event for infection may occur from the date of fracture to 90 days after the definitive fracture management surgery **AND** involves deep soft tissues of the incision (e.g., fascial and muscle layers) **AND** patient has at least one of the following: a. purulent drainage from the deep incision. b. a deep incision that spontaneously dehisces, or is deliberately opened or aspirated by a surgeon, attending physician or other designee, and organism is identified by a culture or non-culture based microbiologic testing method which is performed for purposes of clinical diagnosis or treatment (e.g., not Active Surveillance Culture/Testing [ASC/AST]) or culture or non-culture based microbiologic testing method is not performed **AND **patient has at least one of the following signs or symptoms: fever (> 38 °C); localized pain or tenderness. A culture or non-culture-based test that has a negative finding does not meet this criterion. c. an abscess or other evidence of infection involving the deep incision that is detected on gross anatomical or histopathologic exam, or imaging test
*Organ/Space SSI*	Date of event for infection may occur from the date of fracture to 90 days after the definitive fracture management surgery **AND** infection involves any part of the body deeper than the fascial/muscle layers, that is opened or manipulated during the operative procedure **AND** patient has at least one of the following: a. purulent drainage from a drain that is placed into the organ/space (e.g., closed suction drainage system, open drain, T-tube drain, CT guided drainage) b. organisms are identified from an aseptically obtained fluid or tissue in the organ/space by a culture or non-culture based microbiologic testing method which is performed for purposes of clinical diagnosis or treatment (e.g., not Active Surveillance Culture/Testing [ASC/AST]). c. an abscess or other evidence of infection involving the organ/space that is detected on gross anatomical or histopathologic exam, or imaging test evidence suggestive of infection. **AND** meets at least one criterion for a specific organ/space infection site summarized in the Surveillance Definitions for Specific Types of Infections chapter.^1^

*The CDC criteria have been modified to include all definitive fracture management surgeries instead of including only National Healthcare Safety Network procedures that require infection reporting

#### Secondary outcome

The secondary outcome is the occurrence of an unplanned fracture-related reoperation within 12 months of the fracture. Unplanned reoperations are a common, patient-important outcome in fracture surgery research that captures severe wound and bone healing complications that may be related to occult infections [6, 15, 16]. Our definition includes treatments for infection, wound healing complications, or fracture healing complications such as a delayed union or nonunion. We will also report the occurrence of each type of unplanned reoperation by treatment arm.

### Analysis methods

We will report the number and percentage of patients who sustain the study outcomes by treatment group. We will evaluate the effect of the preoperative antiseptic solutions on our study outcomes using mixed effects regression models with a binomial distribution to produce treatment effect estimates presented as odds ratios with 95% confidence intervals as recommended (Table 2) [17]. As suggested by Morgan et al. and Hemming et al., we will include time and treatment as fixed effects and use random effects to account for the complex correlation structure [18–20]. We will consider three correlation structures, in the following sequence: exponential decay, nested exchangeable, and exchangeable. If we experience convergence issues or find insufficient between-period correlation to support an exponential decay or nested exchangeable structure, we will assume an exchangeable correlation structure. The models will also include prespecified covariates prognostic of infection or unplanned reoperation as fixed effects. These covariates are the severity of the open fracture, location of the fracture, and severity of the wound contamination [21]. The same covariates will be used for all primary and secondary outcomes. This planned analysis is a more complex structure than we proposed in the initial study protocol but represents the most recently recommended statistical techniques for cluster-crossover trial analysis [18, 20, 22, 23]. Estimated intracluster correlation coefficients will also be reported [24].

**Table 2:** Study outcomes

	Povidone-iodine (***n*** = XXX)	Chlorhexidine (***n*** = XXX)	Odds ratio (95% CI)	ARR (95% CI)	***p*** value
*Primary outcome*					
Surgical site infection					
Superficial infection by 30 days					**–**
Deep incisional by 90 days					**–**
Organ/space infection by 90 days					**–**
*Alternative definitions of SSI*					
Any surgical site infection by 365 days					
Fracture-related infection by 365 days					
*Secondary outcome*					
Unplanned reoperation by 365 days					
Unplanned reoperation for infection by 365 days					**–**
Unplanned reoperation for wound healing complications by 365 days					**–**
Unplanned reoperation to promote fracture healing by 365 days					**–**

Our primary and secondary analyses will use multiple imputations to account for missing data. The multiple imputation analysis will create 100 imputed datasets using multivariate imputation by chained equations and pooled using Rubin’s rules for combining [25]. The imputation will be performed separately within each treatment arm.

### Subgroup analyses

To determine treatment effect heterogeneity on the study outcomes, we will use the same analytical approach as specified for the primary and secondary outcomes above but include a treatment by subgroup interaction term in the model. We will report results by the prespecified subgroups, which consists of the severity of the open fracture (Gustilo-Anderson type I or II versus type III), upper extremity versus lower extremity open fractures, and the severity of the wound contamination (none, minimal, or surface contamination versus embedded wound contamination) using a forest plot reporting odds ratios with 95% confidence intervals. These analyses will be approached and reported in accordance with best practices and guidelines for subgroup analyses [26–30]. We will use the criteria suggested by Schandelmaier et al. to guide inferences about the credibility of our subgroup analyses [30]. As participants may have more than one included fracture representing different subgroups; the analyses will be performed by categorizing participants according to the fracture with the most severe injury characteristic for each subgroup.

### Sensitivity analyses

We will consider four alternative analysis approaches to evaluate the robustness of our findings, including an alternative definitions of the primary outcome, an as-treated analysis of the primary and secondary outcomes, a complete case missing data analysis of the primary and secondary outcomes, and a Bayesian analysis of the primary and secondary outcomes. We will also allow for post hoc sensitivity analysis based on information not anticipated in advance.

#### Alternative definitions of SSI

To evaluate the robustness of the result, we will consider two alternative exploratory definitions of SSI: (1) using the confirmatory criteria from the consensus definition of fracture-related infection (FRI) and (2) expanding the CDC criteria for all types of SSI to within 1 year of injury [31].

Our adjudication of fracture-related infection is defined by the confirmatory criteria outlined in its 2018 consensus definition [31]. The FRI criteria have been selected as an exploratory outcome because the CDC criteria have been criticized for failing to adequately account for the complexities of infections in traumatic fractures [31, 32]. The FRI criteria attempt to improve the ability to detect infections specifically in fracture patients; however, this definition of FRI has not been fully validated or widely adopted.

The confirmatory criteria include the presence of one or more of the following signs/symptoms:

1. Fistula, sinus, or wound breakdown (with communication to the bone or the implant).
2. Purulent drainage from the wound or presence of pus during surgery.
3. Phenotypically indistinguishable pathogens identified by culture from at least two separate deep tissue/implant (including sonication-fluid) specimens taken during an operative intervention. In the case of tissue, multiple specimens (3) should be taken, each with clean instruments (not superficial or sinus tract swabs). In cases of joint effusion arising in a joint adjacent to a fractured bone, fluid samples obtained by sterile puncture may be included as a single sample.
4. Presence of microorganisms in deep tissue taken during an operative intervention, as confirmed by histopathological examination using specific staining techniques for bacteria or fungi.

The second exploratory definition of surgical site infection expands the CDC criteria to a 12-month surveillance period. This outcome will use the same diagnostic CDC reporting criteria for the primary; however, the timeframe for this outcome will be expanded to include all SSIs that occur within 12 months of open fracture. Similar to the rationale for using the FRI outcome and the recommendations for a minimum of 12-month follow-up for orthopedic fracture outcomes, this expanded timeframe will detect infections that occur beyond the standard CDC surveillance reporting periods. This modification of the CDC reporting periods has been used in previous orthopedic fracture trials [6, 33].

#### As-treated analysis

One of our sensitivity analyses will be performed on an as-treated population. The as-treated population will include participants from the intention-to-treat population who received one of the two interventions; however, participants will be classified based on the intervention received at their definitive fracture management surgery. Participants who do not receive one of the study interventions will be removed from this analysis. Similar to the primary analysis, we will use mixed effects regression models with a binomial distribution and the same covariates and correlation structure as the primary model. A more simplified structure will be considered if we encounter convergence issues with this model.

#### Missing data

While we anticipate minimal missing outcome data, we will perform a sensitivity analysis on the primary and secondary analyses to explore the impact of missing outcome data. Our sensitivity analysis will be a complete case analysis, including only those patients with a known status of the outcome being analyzed.

#### Bayesian analysis

The Bayesian analyses will be performed using four different priors (neutral with moderate strength, neutral flat, optimistic with moderate strength, and pessimistic with moderate strength) defined on a log-odds scale and described below. The neutral priors will be centered on a log odds of 0 (odds ratio of 1). The neutral flat prior will have a standard deviation of 100. The optimistic prior will be centered on the estimated effect size of a 0.62 odds ratio (log odds of − 0.48). In contrast, the pessimistic prior is centered on the same effect size but for the alternative treatment. As suggested by Zampieri et al. [34], the standard deviation of 0.48 was selected for the moderate strength priors as it allows for a 15% probability of the alternative treatment benefit in both the optimistic and pessimistic prior. The prior probability of our neutral prior with a moderate strength distribution implies a 68% chance the estimated effect will be between an odds ratio of 0.62 and 1.38. The neutral prior with moderate strength will be our preferred prior in this sensitivity analysis.

The modeling for the Bayesian analysis will be consistent with our primary analysis. We will use a mixed effects regression model with a Bernoulli distribution. The model will include time and treatment as fixed effects and use random effects to account for the complex correlation structure. The best fitting correlation structure will be determined using information criteria. If we experience convergence issues with this model structure, we will transition to a less complex model.

**Priors** **used** **in the analysis with their interpretation and a visual depiction.**


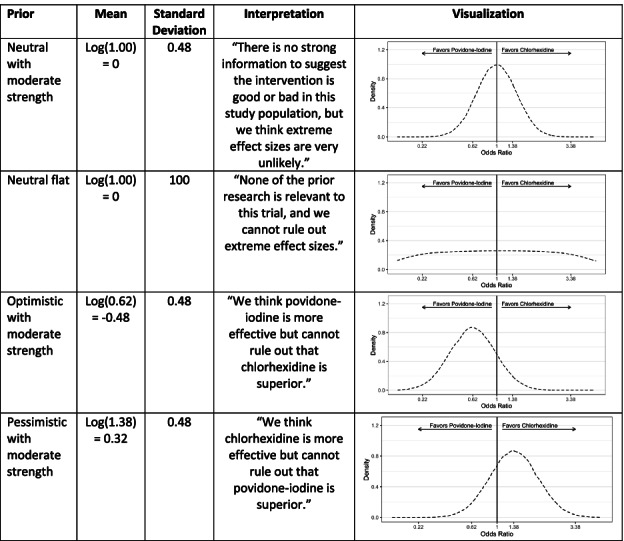


### Harms

The number and percentage of patients experiencing serious adverse events will be presented by treatment arm. No formal statistical testing will be undertaken.

### Statistical software

The statistical analyses will be performed with SAS, version 9.4 (SAS Institute, Cary, NC) and R (R Foundation for Statistical Computing, Vienna, Austria).

## Data Availability

The datasets generated and/or analyzed during the current study are not publicly available due to the trial still being ongoing, but will be available from the corresponding author on reasonable request.

## Abbreviations

ASC/AST: Active surveillance culture/testing
Aqueous-PREP: A Pragmatic Randomized trial Evaluating Pre-operative aqueous antiseptic skin solutions in open fractures
BMI: Body mass index
CDC: Centers for Disease Control
FRI: Fracture-related infection
OTA-OFC: Orthopedic Trauma Association open fracture classification
ST: Soft tissue
SSI: Surgical site infection
